# Treatment of Pneumonia

**Published:** 1881-08-20

**Authors:** T. B. Greenley

**Affiliations:** Kentucky


### TREATMENT OF PNEUMONIA.

BY T. B. GREENLEY, M.D., OF KENTUCKY.

I was much gratified by the reading of Dr. Word's remarks on the treatment of pneumonia in the June number of the RECORD. I entirely accord with his ideas in that regard. I was also much pleased with the article of Dr. Townsend on the same subject, in the April number of the RECORD, whose views are very similar to Dr. Word's. I believe the treatment, as recommended in those two articles, embrace the therapeutics of pneumonia now in general practice. I have observed for some time on the part of a few practitioners, that there is considerable effort making to restore the old antiphlogistic plan of treatment in that disease; even going so far as to recommend copious blood letting. Now it had been hoped that the days of *phlogiston* had long since measurably departed never to return; but, as some philosopher long since remarked that everything takes place in cycles, and as the free blood-letting system abated thirty years ago, it is perhaps time for its return.

Prof. Gross now stands foremost in the ranks of those who advocate blood-letting in pneumonia; and it is to be greatly regretted that he does, as his great name and influence will tend to give much impetus to the spread of the practice.

I have been practicing medicine thirty-six years and am confident that in my early practice I have seen patients bled, purged, vomited and starved to death, in order to prevent their dying from inflammation and fever. This was called the *antiphlogistic plan.* In my student days it was a familiar thing to see a patient brought before the class at the hospital, ordered to be bled so many ounces from the arm, and so much by wet cups over the chest; also a purgative with antimony, at regular intervals, to control the fever. As a general thing, at our next visit, it would be announced to us that the patient before us at the last meeting was dead.

I have always regarded pneumonia as a graded disease, and requiring treatment accordingly. When only one lobe, say of the right lung, is involved, in a subject of previous good health, but little treatment is necessary; and that mainly should be, if possible, to prevent the further extension of the disease. It is impossible to say what the proper treatment should be in pneumonia unless we have a given case. We might have cases on hand at the same time requiring exactly reverse treatment.

The stout, healthy man of temperate habits would require depressing treatment, while the drunkard or previously debilitated subject would need the supporting plan. Hence we cannot prescribe any special plan of management unless we have some knowledge of the health and vitality of the patient before the attack.

Now the question arises, can we, in a case of previous robust health, reduce the power of the circulation so as to control the disease, and, if possible, prevent its further extension, as well and safely by medication, as we can by venesection? Or if the same result can be obtained by the former, as by the latter means, which course is the more preferable? And which would be the more likely to hasten or retard convalescence? I leave the reader to answer. I think, without question, we have in our control the action of the heart and arteries just as efficiently by the remedies Dr. Word advises, to wit.: veratrum viride, aconite, gelsemium, etc., as can be done by the lancet. If this proposition be admitted, then why not save the patient's blood and thereby his strength for the period of convalescence? It is said that the blood is the life and strength of the animal; then let us on conservative principles, husband as much as possible these essential elements in the treatment of diseases.

I will admit that a case might be instanced wherein venesection with a full stream to the amount of a few ounces would be perhaps safe and advisable. Were I called to see a patient in the first stage of pneumonia, stricken down in robust health, with deep congestion of the organs, hurried and impeded respiration, full, bounding pulse and flushed face, I would not hesitate to bleed him for momentary relief, until I could bring him under the influence of depressing agents. But a case of this description is rarely presented.

Many years ago one reason for blood-letting in inflammation was to reduce the excess of fibrine, and thereby lessen the dangers of the exudative stage from adhesions, etc. We now believe we can fully as well control dangers from this cause by the use of alkalies, such as potassa, ammonia, etc.

In patients of previously broken down health, either from disease or dissipation, it is necessary to use supporting treatment from the outset. Then we can use Prof. Hint's plan of quinine and whisky with benefit.

As a general rule in the treatment of pneumonia it is desirable to keep up free diaphoresis, and nothing that I have tried effects this object better than enveloping the chest with a flax seed meal poultice. It retains its moisture a long time, which obviates the necessity of frequently changing it, thereby affording great advantage over all other poultices. In this state of gentle perspiration, the patient always expresses himself as feeling comfortable; and by this means we reduce the volume of the circulation much more safely than by venesection, and at the same time without exhausting to any great extent the powers of life.

But I did not set down to write an essay on the treatment of pneumonia, but merely to give expression of accordance with the article refered to and that of Dr. Townsend, in preference to the blood-letting plan.

I hope the conservative tendency of the profession in the treatment of disease is so well established at the present day, that the old, heroic practice of salivation and venesection will not soon be revived.

